# Supplementary material for: Control of single-ligand chemistry on thiolated Au25 nanoclusters
Source: Nat Commun. 2020 Oct 30;11:5498. doi: 10.1038/s41467-020-19327-2 (PMC7603303; doi:10.1038/s41467-020-19327-2)
Supplement: Supplementary file 1 — Supplementary Information [file 41467_2020_19327_MOESM1_ESM.pdf]

Supplementary Information

Control of Single-Ligand Chemistry on Thiolated Au_{25} Nanoclusters

Yitao Cao,[1] Victor Fung,[2] Qiaofeng Yao,[1] Tiankai Chen,[1] Shuangquan Zang,[3] De-en Jiang,[2,*] Jianping Xie[1,4,*]

1. Department of Chemical and Biomolecular Engineering, National University of Singapore, 4 Engineering Drive 4, Singapore 117585
2. Department of Chemistry, University of California, Riverside, California 92521, United States
3. Green Catalysis Center, and College of Chemistry, Zhengzhou University, Zhengzhou 450001, China.
4. Joint School of National University of Singapore and Tianjin University, International Campus of Tianjin University Binhai New City, Fuzhou 350207, China

This file includes:

Supplementary Figure 1-11
Supplementary Note 1
Computational details
Supplementary references

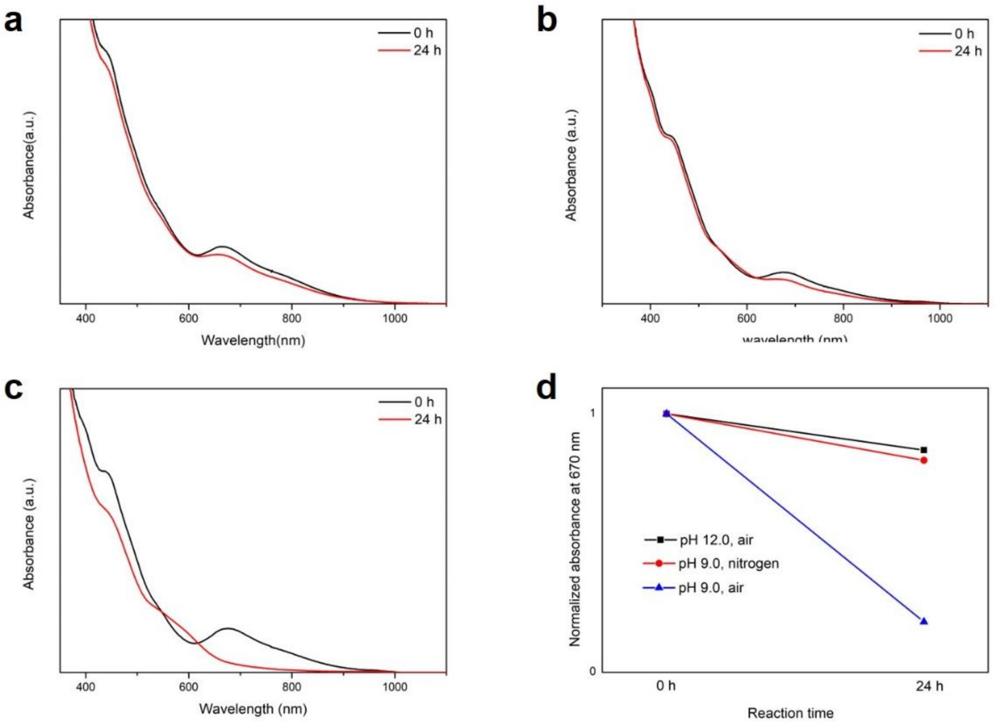

Supplementary Figure 1. Oxidative etching reaction of $[Au_{25}(MHA)_{18}]^-$ monitored by UV-vis absorption spectra at different reaction conditions: (a) reaction at pH of 12.0 in air; (b) reaction at pH of 9.0 after bubbling N_2 for 2 min and kept airtight; (c) reaction at pH of 9.0 in air. (d) The absorbance changes at 670 nm after 24 h reaction under different reaction conditions. The lowered pH and air atmosphere greatly accelerate the oxidative etching reaction.

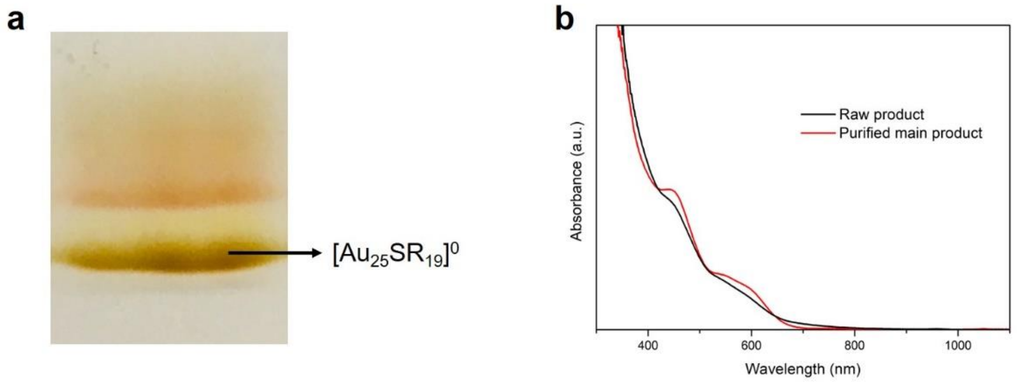

Supplementary Figure 2. (a) Digital photo of bands in PAGE of the raw product of oxidative etching reaction. The band of main product was separated and characterized to be $[Au_{25}MHA_{19}]^0$. (b) UV-vis absorption spectra of the raw product and the separated main product. The absorption features become more distinct after purification.

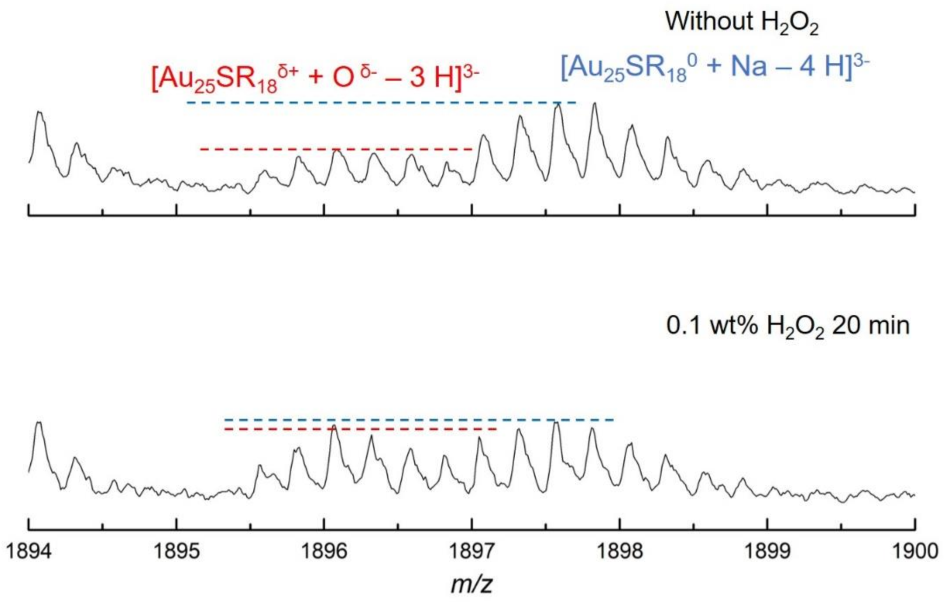

Supplementary Figure 3. Signals corresponding to $[Au_{25}MHA_{18}^{\delta+} + O^{\delta-}]$ adducts become stronger after introducing excess H_2O_2.

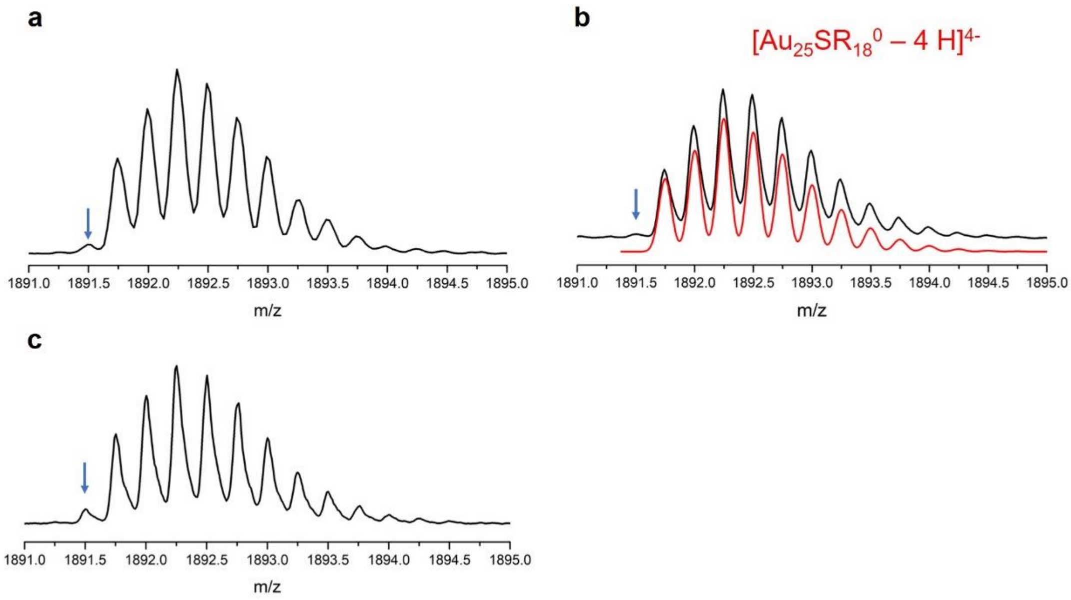

Supplementary Figure 4. (a) Isotopic peaks of [$Au_{25}MHA_{18}$] species after prolonged exposure in air. The intensity of signals corresponding to +1 state species didn't show obvious increase and the majority of [$Au_{25}MHA_{18}$] species was still in 0 state. (b) Signals corresponding to +1 state species were suppressed after bubbling CO. (c) Signals corresponding to +1 state species were increased after introducing H_2O_2.

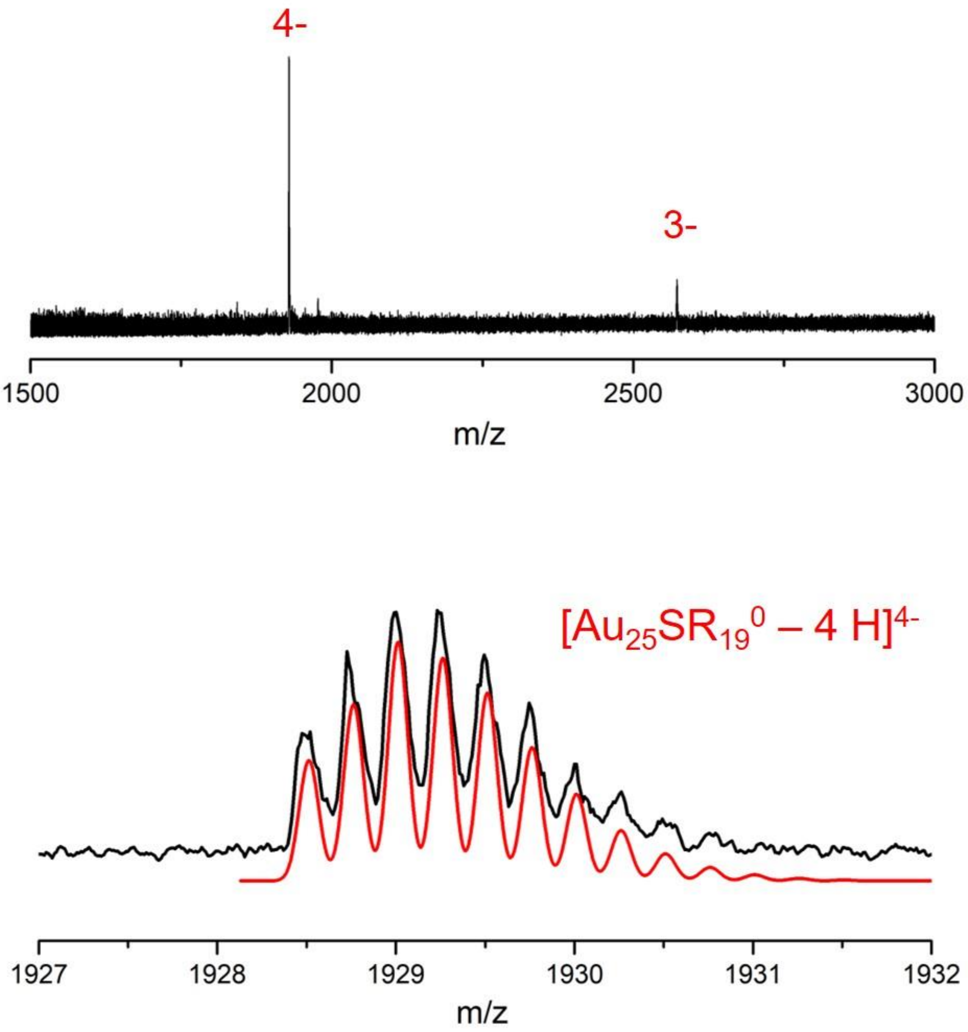

Supplementary Figure 5. ESI-MS spectra of final product obtained by reaction between [$Au_{25}MHA_{18}$] species and excess thiol ligands. The final product was identified as $[Au_{25}MHA_{19}]^0$ with high purity based on the overall spectrum and isotopic analysis.

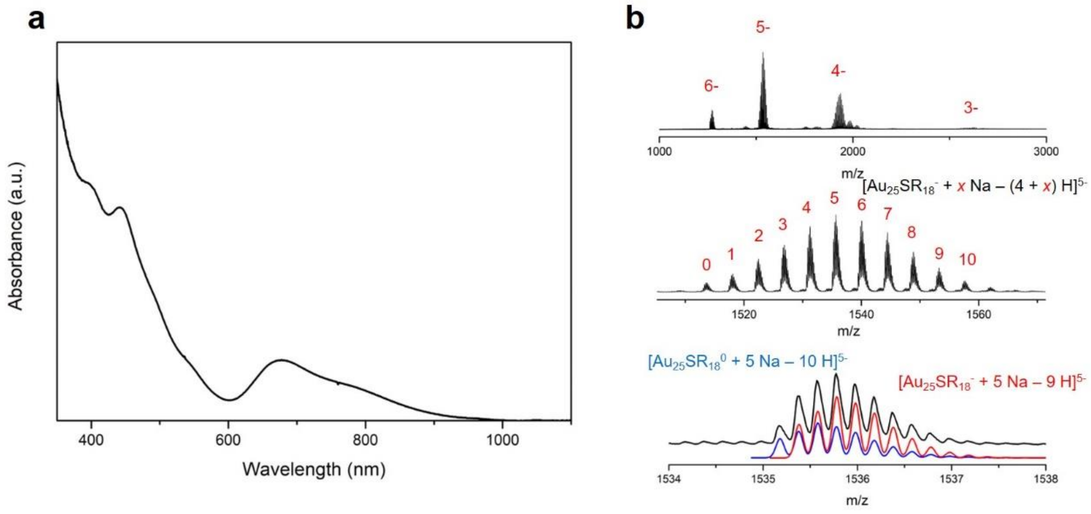

Supplementary Figure 6. (a) UV-vis absorption spectrum and (b) ESI-MS spectrum of $[Au_{25}MHA_{18}]^{-}$ obtained by CO-reduction of $[Au_{25}MHA_{19}]^{0}$. The absorption features shown in UV-vis absorption spectrum and the perfect correspondence of the experimental and simulated signals in ESI-MS spectrum clearly indicate the high purity of $[Au_{25}MHA_{18}]^{-}$.

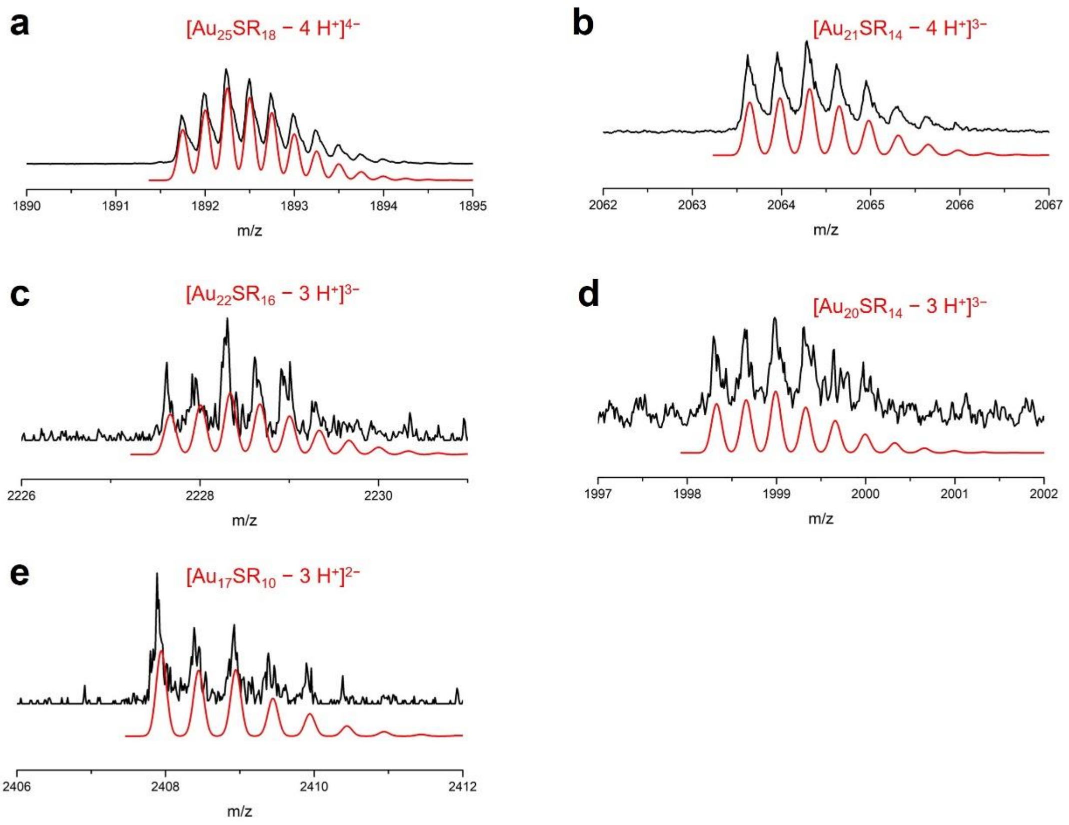

Supplementary Figure 7. Enlarged ESI mass spectra of fragment species of $Au_{25}MHA_{18}$. (a) $Au_{25}MHA_{18}$, (b) $Au_{21}MHA_{14}$, (c) $Au_{22}MHA_{16}$, (d) $Au_{20}MHA_{14}$, and (e) $Au_{17}MHA_{10}$. Both experimental isotopic patterns and their corresponding simulated results were shown.

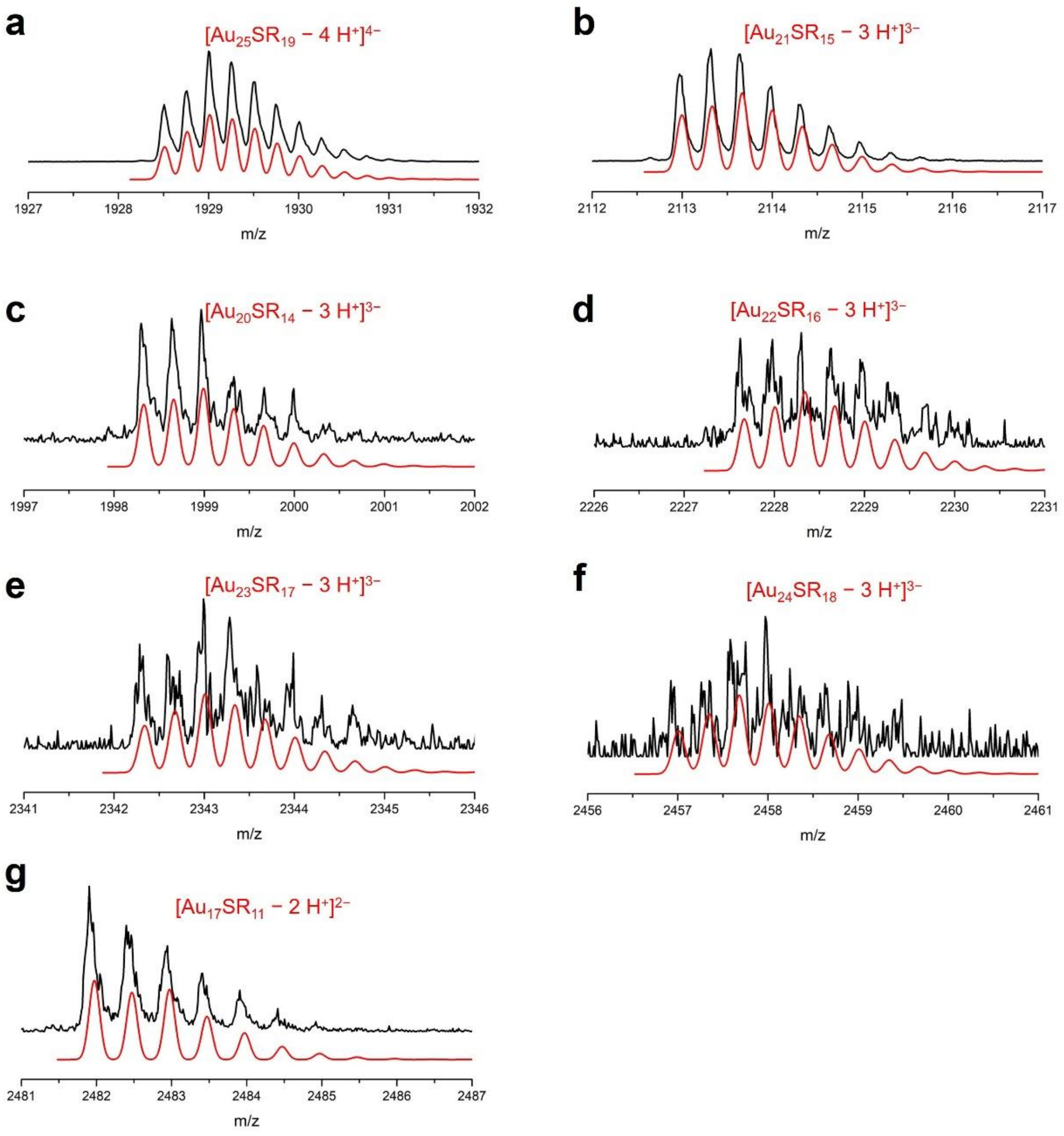

Supplementary Figure 8. Enlarged ESI mass spectra of fragment species of $Au_{25}MHA_{19}$. (a) $Au_{25}MHA_{19}$, (b) $Au_{21}MHA_{15}$, (c) $Au_{20}MHA_{14}$, (d) $Au_{22}MHA_{16}$, (e) $Au_{13}MHA_{17}$, (f) $Au_{24}MHA_{18}$, and (g) $Au_{17}MHA_{11}$. Both experimental isotopic patterns and their corresponding simulated results were shown.

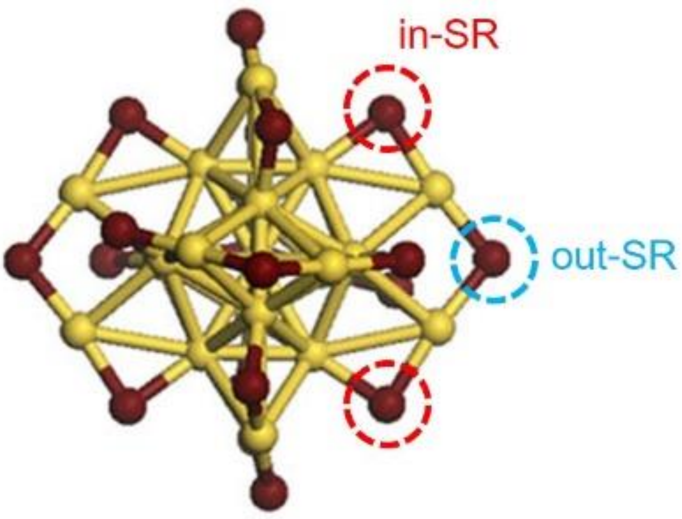

Supplementary Figure 9. Assignment of the two kinds of thiolate ligands in $[Au_{25}SR_{18}]^{-}$, denoted as in-SR and out-SR, respectively.

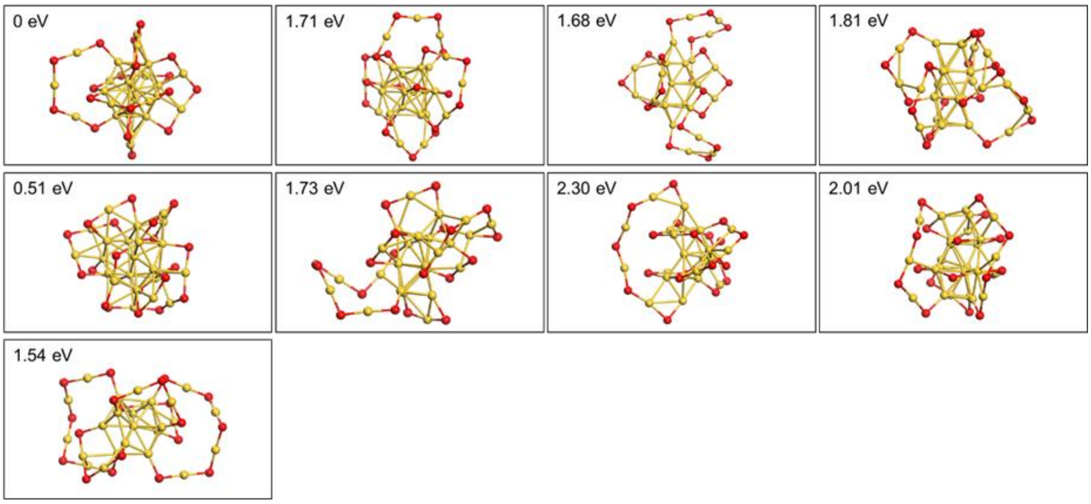

Supplementary Figure 10. Relative energies of the tested geometries for $[Au_{25}SR_{19}]^0$ with the putative global minima used in subsequent calculations defined at 0 eV (at the TPSS/def2-SVP level). Nine different structures were test. The most stable one can be viewed as drawing out one Au atom on the outer shell of Au_{13} core, transforming one Au_2SR_3 staple to Au_3SR_4.

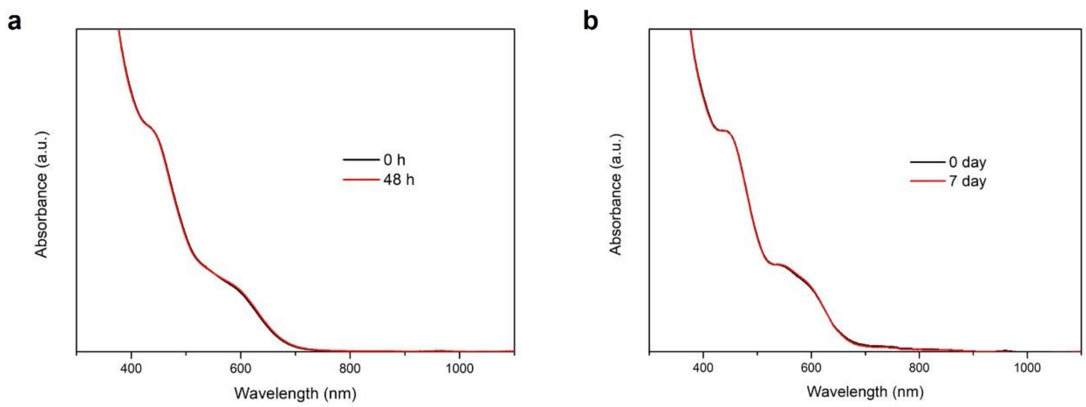

Supplementary Figure 11. (a) UV-vis absorption spectra of $[Au_{25}MHA_{19}]^0$ solution after 2 days in the presence of excess thiol ligands (mole ratio: ligand/cluster = 1/1) in air. (b) UV-vis absorption spectra of $[Au_{25}MHA_{19}]^0$ solution after 7 days in air. No observable changes in its absorption spectra indicates the high stability of $[Au_{25}MHA_{19}]^0$.

Supplementary Note 1.

To understand this phenomenon, we will consider the charge states and corresponding population of $[Au_{25}MHA_{18}]^q$ (q = −1, 0, and +1) species during the reaction. As shown in Fig. 3, after a long-time preserving in air without the addition of excess thiol ligands, most of $Au_{25}MHA_{18}$ species are in 0 state with a small amount of nanoclusters of −1 state (Fig. 3a, a shoulder peak at 800 nm) and +1 state (Fig. 3c). $[Au_{25}MHA_{18}]^0$ is relatively stable in solution and the abundance of $[Au_{25}MHA_{18}]^+$ didn't increase after a long-time preserving, indicating an equilibrium between $[Au_{25}MHA_{18}]^0$ and $[Au_{25}MHA_{18}]^+$ species (Supplementary Fig. 4a). After the introduction of free thiol ligands, $[Au_{25}MHA_{18}]^+$ will rapidly transform to $[Au_{25}MHA_{19}]^0$ and the equilibrium will move toward regeneration of the consumed $[Au_{25}MHA_{18}]^+$. Thus, $[Au_{25}MHA_{18}]^+$ can be regarded as a reaction intermediate with low concentration. In addition, as these $[Au_{25}MHA_{18}]^q$ (q = −1, 0 and +1) species feature very similar absorption spectra,[1] the influence of small amount of $[Au_{25}MHA_{18}]^-$ and $[Au_{25}MHA_{18}]^+$ to the overall absorption spectra of the intermediates during the transformation is marginal. As a result, we observed the changes in the absorption of the reaction solution in Fig. 4a, which indicates the transformation from $[Au_{25}MHA_{18}]^0$ to $[Au_{25}MHA_{19}]^0$. This is also the reason for a quasi-one-to-one transformation process.

Computational details.

Parallel, resolution-of-identity density functional theory (DFT) calculations with the TPSS functional form of the meta generalized gradient approximation (meta-GGA) for electron exchange and correlation[2] with def2-SV(P) basis sets were performed with the quantum chemistry program Turbomole V6.560[3]. Effective core potentials which have 19 valence electrons and include scalar relativistic corrections were used for Au[4]. The Conductor-like Screening Model (COSMO)[5] implemented in Turbomole was used to compute the energies of solvated species in water. For accurate reaction energies, single-point calculations were performed with the def2-TZVP basis sets[6] and the B3LYP functional[7] using the geometries obtained from the previously described method.

Supplementary References

1. Negishi, Y., Chaki, N. K., Shichibu, Y., Whetten, R. L. & Tsukuda, T. Origin of magic stability of thiolated gold clusters: a case study on $Au_{25}(SC_6H_{13})_{18}$. *J. Am. Chem. Soc.* **129**, 11322 (2007).
2. Perdew, J. P., Burke, K. & Ernzerhof, M. Generalized Gradient Approximation Made Simple. *Phys. Rev. Lett.* **77**, 3865 (1996).
3. Ahlrichs, R., Bär, M., Häser, M., Horn, H. & Kölmel C. Electronic structure calculations on workstation computers: The program system turbomole. *Chem. Phys. Lett.* **162**, 165 (1989).
4. Andrae, D., Haeussermann, U., Dolg, M., Stoll, H. & Preuss, H. Energy-adjusted ab initio pseudopotentials for the second and third row transition elements. *Theoretical Chemistry Accounts: Theory, Computation, and Modeling (Theoretica Chimica Acta)* **77**, 123 (1990).
5. Klamt, A. & Schüürmann, G. COSMO: a new approach to dielectric screening in solvents with explicit expressions for the screening energy and its gradient. *Journal of the Chemical Society, Perkin Transactions* **2**, 799 (1993).
6. Schäfer, A., Huber, C. & Ahlrichs R. Fully optimized contracted Gaussian basis sets of triple zeta valence quality for atoms Li to Kr. *J. Chem. Phys.* **100**, 5829 (1994).
7. Lee, C. T., Yang, W. T. & Parr, R. G. Development of the Colle-Salvetti Correlation-Energy Formula into a Functional of the Electron-Density. *Phys. Rev. B* **37**, 785 (1988).
